# Pregnancy is associated with reduced progression of symptomatic adenomyosis: a retrospective pilot study

**DOI:** 10.1186/s12884-023-05956-0

**Published:** 2023-09-04

**Authors:** Daiki Hiratsuka, Erika Omura, Chihiro Ishizawa, Rei Iida, Yamato Fukui, Takehiro Hiraoka, Shun Akaeda, Mitsunori Matsuo, Miyuki Harada, Osamu Wada-Hiraike, Yutaka Osuga, Yasushi Hirota

**Affiliations:** Department of Obstetrics and Gynecology, Graduate School of Medicine, The University of Tokyo, 7-3-1 Hongo, Bunkyo-ku, Tokyo, 113-8655 Japan

**Keywords:** Adenomyosis, Childbirth experience, Magnetic resonance imaging, Pregnancy, Uterine size

## Abstract

**Background:**

Adenomyosis is a common gynecological disease in women of reproductive age and causes various symptoms such as dysmenorrhea and heavy menstrual bleeding. However, the influence of pregnancy on the progression of adenomyosis remains unclear. The insight into whether the size of adenomyosis is increased, decreased, or unchanged during pregnancy is also undetermined. The current study aimed to evaluate the influence of pregnancy in patients with symptomatic adenomyosis.

**Methods:**

This study retrospectively enrolled patients diagnosed with adenomyosis by magnetic resonance imaging between 2015 and 2022 at The University of Tokyo Hospital. Uterine size changes were evaluated by two imaging examinations. In the pregnancy group, the patients did not receive any hormonal and surgical treatments, except cesarean section, but experienced pregnancy and delivery between the first and second imaging examinations. In the control group (nonpregnancy group), the patients experienced neither hormonal and surgical treatments nor pregnancy from at least 1 year before the first imaging to the second imaging. The enlargement rate of the uterine size per year (percentage) was calculated by the uterine volume changes (cm^3^) divided by the interval (years) between two imaging examinations. The enlargement rate of the uterine size per year was compared between the pregnancy group and the control group.

**Results:**

Thirteen and 11 patients with symptomatic adenomyosis were included in the pregnancy group and in the control group, respectively. The pregnancy group had a lower enlargement rate per year than the control group (mean ± SE: −7.4% ± 3.6% vs. 48.0% ± 18.5%, *P* < 0.001), indicating that the size of the uterus with adenomyosis did not change in the pregnancy group.

**Conclusions:**

Pregnancy is associated with reduced progression of symptomatic adenomyosis.

## Background

Adenomyosis is a benign gynecological disease wherein the displacement of the endometrium in the myometrium triggers hypertrophy of the surrounding myometrium, leading to uterine enlargement and subsequently, various symptoms, such as dysmenorrhea and heavy menstrual bleeding [1]. Approximately 20% of reproductive-age women have adenomyosis [2], which often coexists with uterine fibroids and endometriosis [3]. Adenomyosis may also result in pregnancy complications such as miscarriage, preterm birth, fetal growth restriction, preeclampsia, and placental malposition [4–6]. Moreover, the adenomyotic lesion size reportedly degenerates during pregnancy, causing abdominal pain and fever [7–12]. However, the influence of pregnancy on adenomyosis progression remains unclear. The insight into whether the size of adenomyosis is increased, decreased, or unchanged during pregnancy is also undetermined. Thus, this single-center retrospective study aimed to investigate the effect of pregnancy on uterine size in women with symptomatic adenomyosis.

## Methods

### Data collection

This study was performed in accordance with the Declaration of Helsinki and the Ethical Guidelines for Medical and Biological Research Involving Human Subjects formulated by the Japanese government. This study was reviewed and approved by the Research Ethics Committee of the Faculty of Medicine of the University of Tokyo (IRB number: 3128). Informed written consent was substituted by the informed opt-out procedure because of the retrospective nature of the study. The information about this study was posted on the website of the University of Tokyo Hospital to give participants the opportunity to opt out, and those who did not opt out were considered to have provided tacit consent for study participation. Anonymous clinical data were used for the analysis, and individuals cannot be identified based on the data presented. The waived written consent and the informed opt-out procedure were approved by the Research Ethics Committee of the Faculty of Medicine of the University of Tokyo.

This study retrospectively analyzed the deidentified medical records of 816 patients diagnosed with symptomatic adenomyosis through magnetic resonance imaging (MRI) between January 2015 and June 2022 at the University of Tokyo Hospital. Adenomyosis was characterized by high-signal myometrial foci on T2- or T1-weigh sequences, junctional zone (JZ) thickness greater than 12 mm, focal relative thickening of JZ, or poorly defined JZ boundaries [13]. Among these adenomyosis patients, those who underwent two or more MRIs were selected in this study. The first MRI was performed for all patients with symptomatic adenomyosis. The second MRI was performed for the reassessment of adenomyosis in patients with worsened symptoms or infertility, and for preconceptional reevaluation of adenomyosis in patients with a history of pregnancy complications such as miscarriage, preterm birth, and preeclampsia. The patients were classified into two groups: the pregnancy group and the control (nonpregnancy) group. The pregnancy group consisted of patients who became pregnant and delivered between the first and second MRIs. Patients who received any hormonal and surgical treatments, except cesarean section, or who had a miscarriage in the first trimester were excluded from this group. Conversely, those who did not become pregnant between the first MRI and the second MRI comprised the control group. Patients who experienced hormonal or surgical treatments between the first MRI and the second MRI were excluded. Patients who experienced pregnancy within 1 year before the first MRI were also excluded. Clinical data including age, gravidity, parity, and medical and surgical history were obtained from the medical records. Two subtypes of adenomyosis, namely, incipient and advanced types, were classified by MRI. In the incipient type adenomyosis, the lesion is localized in the inner or outer side of the myometrium, and the healthy myometrium is retained in the opposite side. In the advanced type adenomyosis, the lesion involves the whole thickness of the myometrium [14]. In this classification, a lesion involving the entire myometrium is defined as an advanced type, even if the lesion is localized to the anterior or posterior myometrium. The uterine size is often larger in the advanced type than in the incipient type [14].

### Calculation of the enlargement rate of the uterine size per year

Measuring the size of adenomyosis is originally difficult because the boundary between the normal myometrium and the lesion is ambiguous. Therefore, we employed the measurement of uterine size as an alternative method to evaluate the progression of adenomyosis [14]. We used MRI for uterine measurement because this tool is less likely to make differences among examiners compared with ultrasonography and is relatively better at evaluating the correlation of adenomyosis with symptoms [15]. The size of the entire uterus was estimated by using the ellipsoid formula: anteroposterior diameter × longitudinal diameter × transverse diameter × 0.5236 [14, 16–18]. For each group, the enlargement rate of the uterine size per year (percentage) was calculated by the uterine volume changes (cm^3^) divided by the interval (years) between two MRI examinations. The enlargement rate of the uterine size per year was compared between the pregnancy group and the control group. We also measured the myometrial thickness at the adenomyotic site to investigate the influence of pregnancy on the size of the adenomyotic lesion. In the patients with concurrent leiomyomas, the diameter of the largest leiomyoma in each patient was measured. In addition, the pregnancy group was further divided into two subgroups: size-reduction subgroup and nonreduction subgroup. The enlargement rate of the uterine size per year was − 5% or less in the size-reduction subgroup and was more than − 5% in the nonreduction subgroup.

### Statistical analysis

All statistical data were analyzed using Excel. Two groups were compared using Fisher’s exact test and Mann–Whitney *U* test. Furthermore, a *P*-value of less than 0.05 was considered statistically significant.

## Results

### Patient characteristics of the pregnancy and control groups

**Fig. 1:**
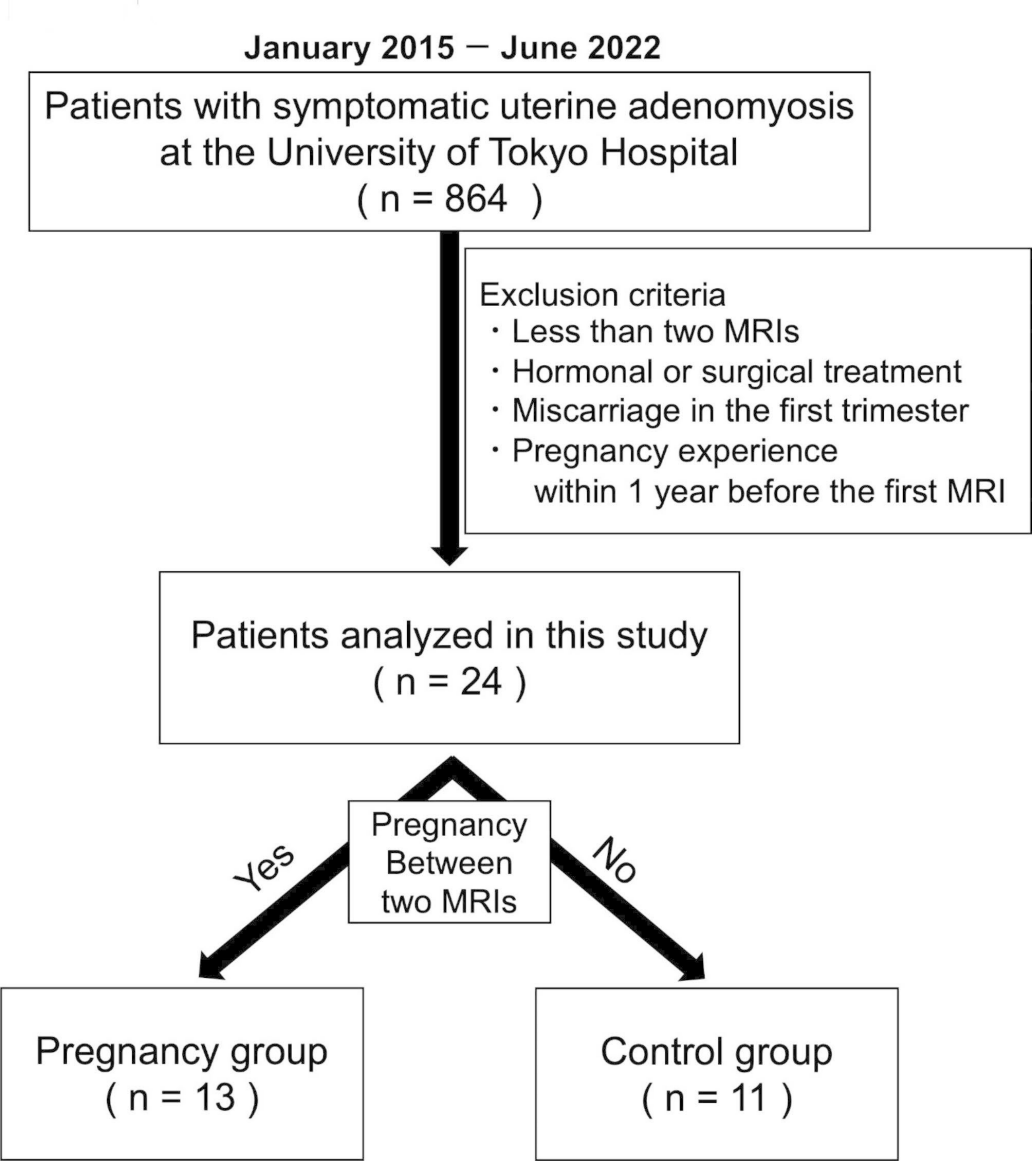
The flowchart of the study

Figure 1 shows the flowchart of this study. Out of 806 patients with symptomatic adenomyosis being reviewed, 13 were included in the pregnancy group, and 11 in the control group. Other than parity and surgery history after the second MRI, the clinical backgrounds were not significantly different between the two groups (Table 1). Leiomyomas were present in some cases in both groups, but the enlargement rate of leiomyoma was not significantly different between the two groups (*P* = 0.122). Surgical treatment after the second MRI was provided to 31% (4/13) of patients in the pregnancy group and 73% (8/11) in the control group (*P* = 0.100). All surgeries performed on patients in the pregnancy group were hysterectomy (4/4). On the other hand, 50% in the control group were hysterectomy (4/8) and the other 50% were adenomyomectomy to preserve fertility (4/8). Table 2 lists the clinical features of each patient in the pregnancy group. To conceive, 62% (8/13) of the patients underwent in vitro fertilization and embryo transfer (IVF-ET). Regarding the mode of delivery, 84% (11/13) of the pregnant patients underwent cesarean section. In the control group, 45% (5/11) underwent fertility treatment or had a desire to conceive.

**Table 1:** Characteristics of patients with adenomyosis in the pregnancy group and the control (nonpregnancy) group. MRI was used to evaluate the presence or absence of endometriosis and leiomyoma and to classify the two adenomyosis subtypes (incipient and advanced types). The incipient-type adenomyosis is localized in the inner or outer side of the myometrium, while the healthy myometrium is retained in the opposite side. The advanced-type adenomyosis involves the entire myometrium. MRI, magnetic resonance imaging; SD, standard deviation; SE, standard error

Index	Pregnancy group	Control (nonpregnancy) group	*P*-value
Age at 1st MRI (mean ± SD)	34.9 ± 2.6	38.5 ± 5.5	0.147
Age at 2nd MRI (mean ± SD)	38.9 ± 3.2	41.5 ± 5.2	0.246
Interval of the two MRI examinations (y, mean ± SD)	4.0 ± 3.0	3.1 ± 2.5	0.192
Parity (mean ± SD)	1.2 ± 0.4	0.2 ± 0.4	< 0.001
History of dilation and curettage	54% (7/13)	45% (5/11)	1.000
Endometriosis	54% (7/13)	27% (3/11)	0.296
Leiomyoma	54% (7/13)	64% (7/11)	0.725
Diameter of the largest leiomyoma (1st MRI) (cm, mean ± SD)	2.0 ± 0.8	2.6 ± 1.4	0.400
Diameter of the largest leiomyoma (2nd MRI) (cm, mean ± SD)	2.1 ± 1.1	3.5 ± 2.4	0.172
Enlargement rate of the largest leiomyoma per year (%, mean ± SE)	-3.4 ± 4.1	5.2 ± 3.2	0.122
Subtype of adenomyosis	Incipient, 23% (3/13); Advanced, 77% (10/13)	Incipient, 45% (5/11); Advanced, 55% (6/11)	0.272
Uterine size (1st MRI) (cm^3^, mean ± SD)	216.3 ± 119.6	328.2 ± 224.9	0.111
Uterine size (2nd MRI) (cm^3^, mean ± SD)	186.2 ± 109.8	622.5 ± 519.5	0.001
Enlargement rate of the uterine size per year (%, mean ± SE)	-7.4 ± 3.6	48.0 ± 18.5	< 0.001
Myometrial thickness at the adenomyotic site (1st MRI) (cm, mean ± SD)	3.5 ± 1.1	4.3 ± 1.6	0.170
Myometrial thickness at the adenomyotic site (2nd MRI) (cm, mean ± SD)	3.4 ± 1.1	5.7 ± 1.9	< 0.001
Enlargement rate of myometrial thickness at the adenomyotic site per year (%, mean ± SE)	-2.4 ± 1.7	15.1 ± 3.2	< 0.001
Surgery for adenomyosis after 2nd MRI	31% (4/13)	73% (8/11)	0.100
Interval from 2nd MRI to surgery or most recent follow-up visit (y, mean ± SD)	2.0 ± 2.0	0.6 ± 0.5	0.111

**Table 2:** Detailed clinical information of patients in the pregnancy group. MRI was used to evaluate the presence or absence of endometriosis and leiomyoma and to classify the two subtypes of adenomyosis (incipient and advanced types). The incipient-type adenomyosis is localized in the inner or outer side of the myometrium, while the healthy myometrium is retained in the opposite side. The advanced-type adenomyosis involves the entire myometrium. A, anterior wall; CS, cesarean section; D&C, dilation and curettage; GA, gestational age; IVF-ET, in vitro fertilization and embryo transfer; MRI, magnetic resonance imaging; P, posterior wall; VD, vaginal delivery

Case	Age at 1st MRI	Age at delivery	Age at 2nd MRI	Gravida	Para	History of D&C	Endometriosis	Leiomyoma	Subtype of adenomyosis	Mode of conception	GA at delivery (wk)	Mode of delivery	Hysterectomy after 2nd MRI	Interval from 1st MRI to delivery (year)	Interval from delivery to 2nd MRI (year)	Size-reduction group
1	38	39	41	3	2	0	+	+	Advanced	Natural	37	CS	−	1.2	1.2	+
2	34	36	39	3	1	1	+	−	Incipient (A)	IVF-ET	39	VD	−	2.2	3.3	−
3	39	41	41	3	1	2	−	+	Advanced	Natural	17	VD	+	1.4	0.5	+
4	33	34	38	3	1	1	−	−	Advanced	IVF-ET	34	CS	+	1.2	4.0	−
5	39	41	41	3	2	1	−	−	Advanced	Natural	36	CS	−	2.2	0.4	+
6	35	38	39	1	1	0	+	+	Advanced	IVF-ET	36	CS	−	3.2	0.9	−
7	30	32	35	1	1	0	−	+	Advanced	IVF-ET	37	CS	−	2.1	2.1	−
8	34	36	47	1	1	0	+	−	Advanced	IVF-ET	27	CS	+	2.2	10.9	−
9	34	35	36	7	2	4	−	−	Incipient (P)	Natural	32	CS	−	1.1	1.3	+
10	34	37	38	1	1	0	+	−	Incipient (A)	IVF-ET	37	CS	−	2.7	0.5	−
11	34	36	38	4	1	1	+	+	Advanced	Natural	36	CS	−	1.9	1.6	+
12	33	34	35	1	1	0	+	+	Advanced	IVF-ET	40	CS	+	1.4	0.9	+
13	37	38	38	2	1	1	−	+	Advanced	IVF-ET	33	CS	−	0.8	0.4	+

### Pregnancy is associated with the suppression of increase in size of the uterus with adenomyosis

**Fig. 2:**
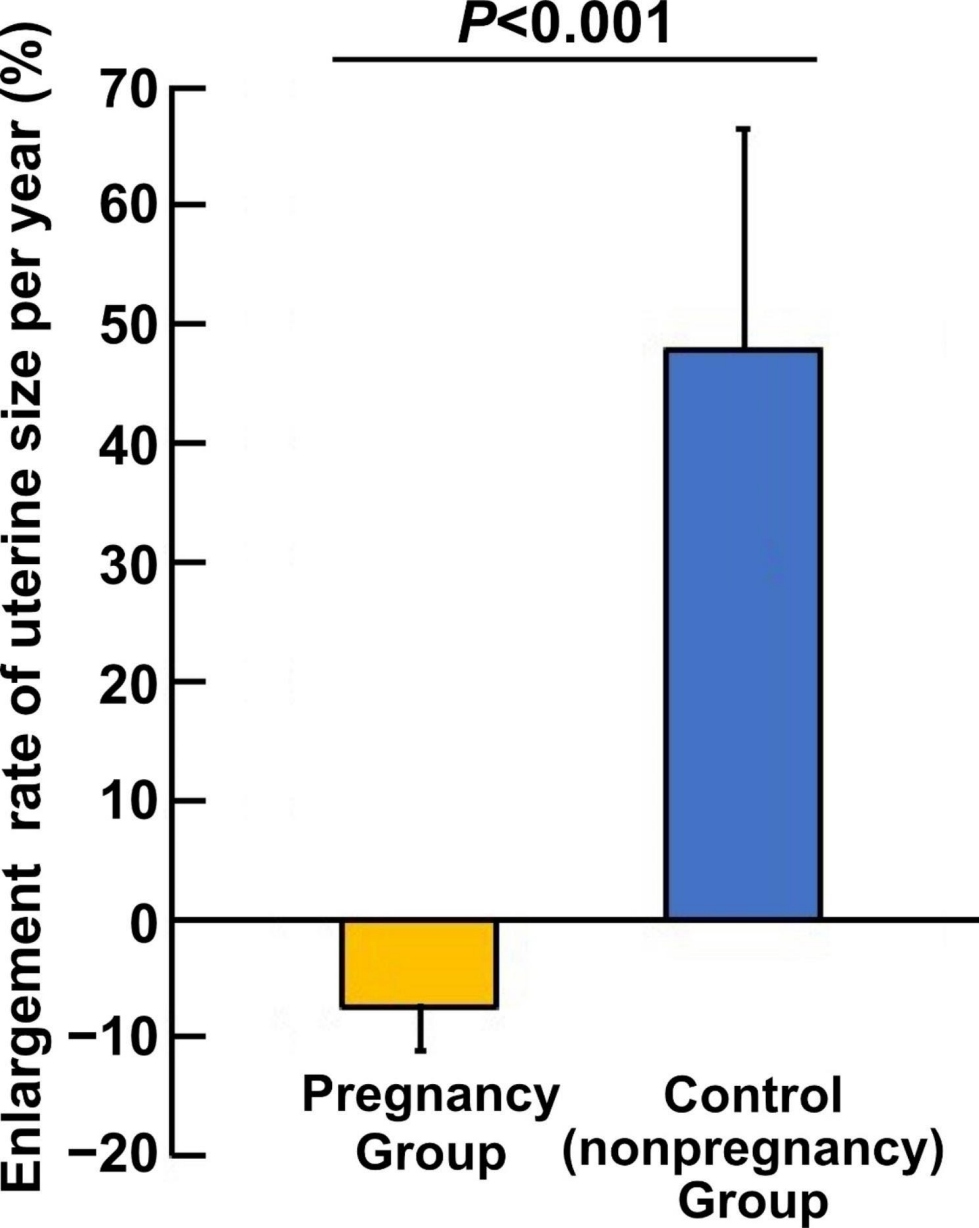
Enlargement rate of the uterine size per year is lower in the pregnancy group. *P* < 0.001, mean ± SEM, Mann–Whitney *U* test

**Fig. 3:**
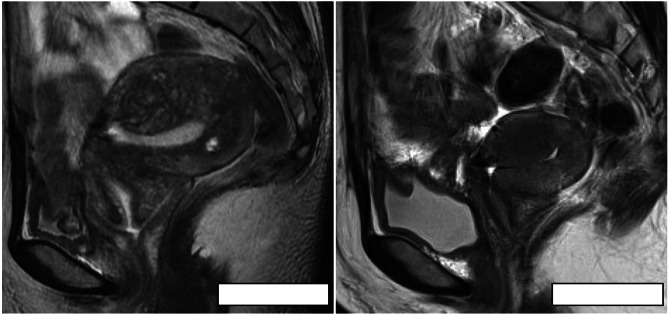
Representative uterine images of T2-weighted MRI before and after pregnancy in a patient with adenomyosis. The left and right images represent the status of adenomyosis before and after pregnancy, respectively. Scale bar = 5 cm. T2WI, T2-weighted image

The enlargement rate of the uterine size per year was − 7.4% ± 3.6% and 48.0% ± 18.5% in the pregnancy and control groups, respectively (*P* < 0.001) (Fig. 2), indicating that the uterine size increases according to the natural course of adenomyosis. The reduced growth of myometrial thickness at the adenomyotic site was also observed in the pregnancy group compared to the control group (− 2.4% ± 1.7% vs. 15.1% ± 3.2%, *P* < 0.001). As shown in Fig. 3, which presents the representative MRI images before and after pregnancy, the adenomyotic lesion shrank and the uterine size decreased markedly after pregnancy. These findings suggest that pregnancy may be involved in the suppression of increase in the size of the uterus with adenomyosis.

### Uterine size increases time-dependently even in patients with adenomyosis with childbirth experience

With regard to the two subgroups of the pregnancy group, 7 were included in the size-reduction subgroup and 6 in the nonreduction subgroup. The enlargement rate of the uterine size per year was defined as − 5% or less in the size-reduction subgroup and was more than − 5% in the nonreduction subgroup. The gestational age at delivery, original uterine size, coexisting endometriosis and leiomyoma, and the subtypes of adenomyosis were not significantly different between the two groups (Table 3). However, IVF-ET was performed more frequently in the nonreduction subgroup than in the size-reduction subgroup (100% vs. 29%, *P* = 0.016), indicating that the fertility condition is related to the change in uterine size. The nonreduction subgroup also had significantly longer intervals from the first MRI to pregnancy (*P* = 0.046) and between the two MRI examinations (*P* = 0.004) and tended to have a longer interval from delivery to the second MRI (*P* = 0.063) than the size-reduction subgroup; thus, the uterine size may increase in a time-dependent manner even in the patients with adenomyosis.

**Table 3:** Comparison of clinical features between the size-reduction and nonreduction subgroups in the pregnancy group. The pregnancy group was divided into two subgroups: size-reduction subgroup and nonreduction subgroup. The enlargement rate of the uterine size per year was − 5% or less in the size-reduction subgroup and was more than − 5% in the nonreduction subgroup. The presence or absence of endometriosis and leiomyoma was evaluated by MRI. GA, gestational age; IVF-ET, in vitro fertilization and embryo transfer; MRI, magnetic resonance imaging; SE, standard error

Index	Size-reduction subgroup (n = 7)	Nonreduction subgroup (n = 6)	*P*-value
Age at delivery	37.7 ± 2.8	35.5 ± 2.2	0.199
Mode of conception	IVF-ET, 29% (2/7); Natural, 71% (5/7)	IVF-ET, 100% (6/6); Natural, 0% (0/7)	0.016
GA at delivery (wk)	33.3 ± 7.6	35.6 ± 4.1	0.568
Endometriosis	43% (3/7)	67% (4/6)	0.592
Leiomyoma	71% (5/7)	33% (2/6)	0.286
Subtype of adenomyosis	Incipient, 14% (1/7); Advanced, 86% (6/7)	Incipient, 33% (2/6); Advanced, 67% (4/6)	0.167
Uterine size at 1st MRI (cm^3^)	261.2 ± 142.2	164.0 ± 62.9	0.253
Uterine size at 2nd MRI (cm^3^)	180.4 ± 129.1	192.9 ± 93.8	0.848
Interval from 1st MRI to delivery (y)	1.4 ± 0.5	2.3 ± 0.7	0.046
Interval from delivery to 2nd MRI (y)	0.9 ± 0.5	3.6 ± 3.8	0.063
Interval from 1st MRI to 2nd MRI (y)	2.3 ± 0.7	5.9 ± 3.6	0.004
Enlargement rate of the uterine size (%, mean ± SE)	−17.2 ± 4.1	3.5 ± 2.0	0.003
Surgery for adenomyosis after 2nd MRI	29% (2/7)	33% (2/6)	0.417

## Discussion

This study revealed that the size of a uterus with symptomatic adenomyosis frequently enlarges according to the natural course of the disease, but childbirth may be involved in the suppression of the increased uterine size. In addition, the uterine size often increases in a time-dependent manner even in the adenomyosis patients who experience childbirth in the observation period. Therefore, the growth of adenomyotic lesions may be suppressed transiently through pregnancy experience. Although the effect of adenomyosis on pregnancy has already been extensively reported, the novelty of this study is to analyze the effect of pregnancy on adenomyotic progression by measuring the uterine volume.

Adenomyosis has various adverse effects during pregnancy, including miscarriage, preterm birth, fetal growth restriction, preeclampsia, placental malposition, and increased cesarean section rate [4, 5]. The degeneration of adenomyosis as a pregnancy complication has also been demonstrated in several case reports. These reports suggest that pregnancy-induced inflammation and necrosis occurring in the adenomyotic lesion cause the temporal volume changes, pain, and fever [7–12]. The timing of symptoms by adenomyotic degeneration varies from the early stages to the postpartum period, depending on the case report, and it is not constant. Degeneration during pregnancy is also seen in endometriosis and uterine fibroids, but these lesions do not always shrink through pregnancy [19, 20]. Myometrial contractions and uterine distension during pregnancy was proposed as the cause of microtrauma and inflammatory reactions of uterine adenomyosis [21], but the mechanism behind the degeneration of adenomyosis is still unclear; however, degeneration could be one of the contributing factors to the changes in uterine size before and after pregnancy. Although the mechanism cannot be definitively concluded, one of the most notable findings in our study is that adenomyosis shrank with pregnancy in more than half of our pregnant patients.

Surgery is the option of choice at our institution when medical treatment is ineffective. In both pregnancy and control groups, most of the symptomatic patients were primarily treated with hormonal therapy after the second MRI. Our study revealed that surgery for adenomyosis was less often conducted in patients with childbirth experience than in those without, suggesting that pregnancy may prevent the progression of adenomyosis and ultimately avoid surgery for adenomyosis.

The present study has some limitations. First, the sample size is small. Since MRI is relatively more accurate than ultrasonography for the diagnosis of uterine adenomyosis [15], the analysis in this study was based on MRI. In clinical practice, having two MRI scans without hormone therapy and surgery is rare. For example, this study excluded pregnant patients with adenomyosis who were inserted with a levonorgestrel-releasing intrauterine system before the resumption of postpartum menstruation, without a second MRI. We therefore consider that the exclusion criteria of two MRIs and no hormone therapy may introduce the possibility of selection bias into this case-control study. We will continue to accumulate the clinical data of patients with adenomyosis for further analyses. Second, we did not assess the lactation status of each pregnant woman. By investigating the duration of lactation, we may be able to determine the appropriate time to start hormone therapy for adenomyosis after childbirth. Third, this study lacked information on the menstrual cycle at MRI; since the thickness of the JZ changes with the menstrual cycle, MRI is better to be taken in the proliferative phase [22]. In this study, all 24 cases had high-signal myometrial foci on T2-weigh sequence at the first MRI, suggesting that subtle variations in the JZ by menstrual cycle were not influential for the total size of the uterus with adenomyosis. However, more accurate analyses may be obtained in the future by taking every MRI at the proliferative phase. Fourth, adenomyosis-derived symptoms such as dysmenorrhea and heavy menstrual bleeding were not included as clinical parameters. Evaluation indexes such as the visual analog scale and the pictorial bleeding assessment chart score may be useful in predicting the effect of pregnancy on adenomyotic lesion size and planning the postpartum treatment [23]. Based on the limitations described above, we agree that this is a preliminary pilot study. However, this study showed that uterine size could remain unchanged or decrease after delivery in patients with adenomyosis. This finding is novel and noteworthy because symptomatic adenomyosis often enlarges if the patient is not treated with hormonal therapy. We believe that more accurate and detailed results will be obtained in the future by increasing the sample size and minimizing the effect of selection bias on the results to clarify the relationship of clinical features with the extent of adenomyotic progression in patients with and without childbirth experience.

## Conclusions

Pregnancy is associated with reduced progression of symptomatic adenomyosis.

## Data Availability

All data generated or analyzed during this study are included in this published article.

## Abbreviations

MRI: Magnetic resonance imaging
IVF-ET: In vitro fertilization and embryo transfer
